# Altered gray matter volumes in post-stroke depressive patients after subcortical stroke

**DOI:** 10.1016/j.nicl.2020.102224

**Published:** 2020-02-20

**Authors:** Wenjun Hong, Zhiyong Zhao, Dongmei Wang, Ming Li, Chaozheng Tang, Zheng Li, Rong Xu, Chetwyn C.H. Chan

**Affiliations:** a Department of Rehabilitation Medicine, Nanjing Drum Tower Hospital, The Affiliated Hospital of Nanjing University Medical School, Nanjing, 210008, China; b Key Laboratory for Biomedical Engineering of Ministry of Education, College of Biomedical Engineering & Instrument Science, Zhejiang University, Hangzhou, China; c Department of Radiology, Nanjing Drum Tower Hospital, The Affiliated Hospital of Nanjing University Medical School, Nanjing, China; d State Key Laboratory of Cognitive Neuroscience and Leaning, Beijing Normal University, Beijing, China; e Applied Cognitive Neuroscience Laboratory, Department of Rehabilitation Sciences, The Hong Kong Polytechnic University, Hong Kong; f University Research Facility in Behavioral and Systems Neuroscience, The Hong Kong Polytechnic University, Hong Kong, China

**Keywords:** Post-stroke depression, Subcortical lesion, Magnetic resonance imaging, Gray matter volume, Support vector machine analyze

## Abstract

Stroke survivors are known to suffer from post-stroke depression (PSD). However, the likelihood of structural changes in the brains of PSD patients has not been explored. This study aims to extract changes in the gray matter of these patients and test how these changes account for the PSD symptoms. High-resolution T1 weighted images were collected from 23 PSD patients diagnosed with subcortical stroke. Voxel-based morphometry and support vector machine analyses were used to analyze the data. The results were compared with those collected from 33 non-PSD patients. PSD group showed decreased gray matter volume (GMV) in the left middle frontal gyrus (MFG) when compared to the non-PSD patients. Together with the clinical and demographic variables, the MFG's GMV predictive model was able to distinguish PSD from the non-PSD patients (0•70 sensitivity and 0•88 specificity). The changes in the left inferior frontal gyrus (61%) and dorsolateral prefrontal cortex (39%) suggest that the somatic/affective symptoms in PSD is likely to be due to patients’ problems with understanding and appraising negative emotional stimuli. The impact brought by the reduced prefrontal to limbic system connectivity needs further exploration. These findings indicate possible systemic involvement of the frontolimbic network resulting in PSD after brain lesions which is likely to be independent from the location of the lesion. The results inform specific clinical interventions to be provided for treating depressive symptoms in post-stroke patients.

## Highlights

• PSD patients showed GMV decreases in the left MFG.
• Change of the MFG's GMV is independent from the lesions.
• Hypoactivity in IFG and DLPFC may account for the PSD symptoms.
• PSD patients may have difficulty in understanding and appraising negative emotions.

## 1 Introduction

Damages to the brain due to stroke result in motor, cognitive, psychosocial, and daily living dysfunctions (Cauraugh et al., 2000; van Rijsbergen et al., 2017; Villa et al., 2018). Care for post-stroke survivors imposes heavy financial and psychosocial burdens on families and caregivers as well (Sun et al., 2013). Recent studies revealed at least 30% of stroke survivors suffered from post-stroke depression (PSD) during the first year (Ayerbe et al., 2013). And within five years, the prevalence of PSD was 29% and incidence ranged from 39% to 52% (Ayerbe et al., 2013). The typical symptoms manifested among the PSD patients were depressed mood, anhedonia, loss of energy, decreased concentration, and psychic retardation (Kim, 2016). PSD was found to aggravate physical and cognitive impairments, resulting in increases in level of disability, risk of falls, and even mortality (Paolucci, 2008). PSD was reported to be functional or pathological in nature, with the former associated with lesions and the latter associated with post-lesion neural changes (Ayerbe et al., 2013). However, the neural mechanism underlying the symptoms PSD remains elusive. The theoretical underpinning of the recent adoption of antidepressant drugs and psychotherapy for patients with PSD remains unclear as well (Hackett et al., 2008; Whyte and Mulsant, 2002). In particular, the benefits brought by using antidepressants containing selective serotonin-reuptake inhibitors are not robust (Kim, 2016). This study aims to gain better understanding of the mechanism of PSD by employing an advanced morphometry analysis to explore the relationship between gray matter volume (GMV) and the depressive symptoms among a group of post-stroke patients with subcortical lesions.

Despite PSD patients have been consistently reported having more severe functional decline than non-PSD patients, findings from recent structural brain imaging studies do not seem to corroborate with the between-group differences. Two studies explored differences in the GMV as markers for the treatment effects of PSD patients, and they reported different results. Shi et al. revealed post-treatment decreases in the GMV in the orbitofrontal cortex, anterior cingulate cortex (ACG), primary motor cortex and supplementary motor area, primary/secondary sensory area among PSD patients with frontal lesions (Shi et al., 2017). In contrast, Balaev et al. reported no significant differences in the GMV in these neural substrates before and after the treatment received by the PSD patients (Balaev et al., 2018). It is noteworthy that the patients recruited in Balaev et al.’s study were mixed with cortical and subcortical lesions. Lesions in the cortex could have confounded the results of these studies particularly involving patients with lesions in the frontal regions.

GMV is derived from voxel-based morphometry (VBM) analysis which has been shown as sensitive in reflecting recovery effect related changes in post-stroke patients (Sihvonen et al., 2017; Wang et al., 2018). Changes in GMV of the superior orbital frontal gyrus were significantly correlated with the severity of late-life depressive mood (Droppa et al., 2017). Increase in GMV of the inferior frontal gyrus was associated with a decline of sustained attention due to major depressive disorder (MDD) (Yang et al., 2015). MDD is also associated with a decrease in GMV in the cingulate cortex, prefrontal lobe, temporal and parieto-occipital cortices, basal ganglia, and cerebellum (Grieve et al., 2013).

This study aimed to address the changes in the frontolimbic network among PSD patients as revealed in previous studies using the GMV method. Different from Shi et al. and Balaev et al., this study attempted to reduce the between-subject variability, as well as to use a more robust and accurate method for conducting the data analyses. These are meant to tackle the shortfalls observed in the previous two studies. First, participants of these two studies had rather large variations in the duration after onset (sub-acute and chronic stages) and brain lesions (frontal and parietal lobes). In this study, the post-stroke patients were in the chronic stage (six months or longer) for controlling the possible spontaneous recovery effect (Kwakkel and Kollen, 2013; Stinear and Byblow, 2014) and their lesions were in the subcortical areas for minimizing the possible post-stroke cortical-related reorganizations (Carey et al., 2011, 2016; Luft et al., 2004). Second, both Shi et al. and Balaev et al. used SPM8 software to perform the VBM analyses. The use of Computational Anatomy Toolbox (CAT) within SPM12 can improve the robustness and accuracy of the VBM analyses (Farokhian et al., 2017). First, the CAT adopts the Adaptive Maximum A Posterior (AMAP) technique for segmentation which does not rely on a priori information on the tissue probabilities and hence minimizes the potential biases (http://dbm.neuro.uni-jena.de/cat12/CAT12-Manual.pdf). Second, the normalized template of the CAT is based on the DARTEL template derived from 555 healthy control subjects, which increase its statistical power of the analyses.

We hypothesized that the GMVs of the PSD patients with subcortical lesion would be different from their non-PSD counterpart in the neural hubs mediating emotion and depressive mood, namely anterior cingulate cortex, prefrontal lobe, and primary somatosensory cortex (Balaev et al., 2018; Shi et al., 2017). It was also hypothesized that the changes in GMV in these significant regions would relate to the symptom severity of the PSD patients.

## 2 Materials and methods

### 2.1 Participants

Post-stroke out-patients were recruited to participate in the study by means of putting up posters and distributing leaflets at Nanjing Drum Tower Hospital. The inclusion criteria were: (1) first onset subcortical stroke as confirmed by magnetic resonance imaging; (2) onset ≥ six months; and (3) right handedness before stroke. The exclusion criteria were: (1) any contraindication for receiving MRI; (2) unstable medical conditions such as severe atrial fibrillation; (3) severe aphasia that hindered meaningful communication and testsadministered by Mini-Mental State Examination (MMSE) and National Institute of Health Stroke Scale (NIHSS); and (4) history of depressive mood or major depression. A total of 69 post-stroke out-patients were recruited. Among them, 29 patients were diagnosed as suffering from PSD by one of the two neurologists on the research team. The diagnostic criteria were those set in the Diagnostic and Statistical Manual of Mental Disorders (4th edition) (DSM-IV) and the Hamilton Depression Rating Scale (HAMD; cut off score > 7) (Zhang et al., 2018). Six PSD patients and seven non-PSD patients were excluded because of the non-first-onset, cortical damage, history of depression prior to the stroke, receiving psychotherapy, and/or inability to complete MRI scans. The final sample size was 56 patients with 23 PSD patients (12 left-sided and 11 right-sided lesions, 12•75 ± 5•32 months post-stroke) and 33 non-PSD patients (16 left-sided and 17 right-sided lesion, 10•47 ± 3•99 months post-stroke) (Fig. A. 1, Table A. 1, and Table A. 2). These sample sizes were comparable with those reported in previous structural brain imaging studies on VBM, ranging from 22 to 35 (Shi et al., 2017; Silva et al., 2013; Yuan et al., 2008). The research protocol and implementation were in accordance with the “Declaration of Helsinki”. Written informed consent was obtained from each patient and the ethics approval was obtained from the research committee of thehospital in which the study was conducted.

### 2.2 Data collection procedure

All patients completed four other measures besides HAMD within sevendays before receiving the brain scan, including Body Mass Index (BMI), Modified Barthel Index (MBI), MMSE, and NIHSS for the quantification of general health condition, functional independence, general cognitive functions, and severity of the stroke, repectively (Egorova et al., 2017; Han et al., 2015; Villa et al., 2018). All patients passed the safety for MRI scan including detection for metal implants in their bodies. Before and after entering the scanner, the patients were reminded to keep their head movements to a minimum and breathe in a steady pace. The brain scan took approximately fourminutes to complete.

### 2.3 Instruments

The HAMD measures depressive mood symptoms (Hamilton, 1960) with good sensitivity (0•84; 95% CI 0•75 to 0•90) and specificity (0•83; 95% CI 0•72 to 0•90) for the identification of patients with PSD (Meader et al., 2014). The BMI measures weight in kilograms divided by the square of height in meters. Lower BMI values were found to associate with the diagnosis of PSD (Han et al., 2015). The Chinese version of MBI measures self-care independence. Its validity and reliability is comparable to the original version (Leung et al., 2007). The Chinese version MMSE has similar psychometric properties when compared with the original version developed in 1975 (Bour et al., 2010; Folstein et al., 1975). The NIHSS is a measure of severity of stroke. There is strong evidence of its ability to predict the discriminative power (Zhang et al., 2012) and mortality risk of post-stroke patient (Brott et al., 1989).

### 2.4 MRI data acquisition

Images were acquired with a 3-T MRI scanner (Philips, Ingenia, Netherlands) at Nanjing Drum Tower Hospital. High-resolution T1-weighted images were acquired using a magnetization-prepared rapid gradient echo sequence: sagittal axis, TR/TE/TI = 15/4•76/1100 ms, FOV = 256 × 256 mm^2^, flip angle = 25°, 256 × 256 = matrix, slices = 144, thickness = 1 mm, gap = 0•5 mm.

### 2.5 Voxel-based morphometry analysis

VBM analysis was performed using CAT12 (http://dbm.neuro.uni-jena.de/cat), an extension toolbox of Statistical Parametric Mapping software (SPM12, http://www.fil.ion.ucl.ac.uk/spm/software/spm12).The default settings were used in reference to the CAT 12 toolbox manual (http://dbm.neuro.uni-jena.de/cat12/CAT12-Manual.pdf). The T1 images were spatially registered according to the Montreal Neurological Institute (MNI) template. Whole brain structural data were segmented into white matter, gray matter and cerebrospinal fluid. Bias correction was performed to remove intensity nonuniformities. Segmented images of the gray matter were preserved to assess the amount of volume changes based on spatial registration. The modulated images of the gray matter after bias correction reflected the tissue volumes, which were used for VBM analysis. The total intracranial volume (TIV) was calculated as a covariate for further statistical analyses. Finally, the normalized gray matter images were smoothed using a Gaussian filter (8 mm full-width halfmaximum, FWHM).

### 2.6 Statistical analyses

Lesions of each patient were manually outlined slice by slice in spatially normalized T1-weighted imagesusing the MRIcron (http://www.cabiatl.com/mricro/mricron/index.html) software. All the lesions were identified and verified by a radiologist. The GMV was assessed by using a voxel-wise two-sample *t*-test within a gray matter mask with TIV as a covariate to correct for different brain sizes among the participants. Level of statistical significance was set at *p* < 0.01 at the voxel level and *p* < 0.05 at the cluster level (GRF corrected). The surviving clusters were reported. In regions showing significant between-group difference, Spearman correlation was computed between the mean GMV, and the mean duration after onset and mean scores of HAMD, BMI, MBI, MMSE, and NIHSS.

#### 2.6.1 SVM classification analysis

Besides the GMV, other significant clinical and demographic parameters previously reported influencing PSD were entered into the classification model as feature variables.The training data set had 56 cases with 33 and 23 were classified as PSD and non-PSD based on the results of the HAMD. All the feature variables were normalized and tested for their similarities (removed if pairwise correlations >0.86) and between-group differences. The final feature variables entered into the classification models were age, sex, education level, duration of illness, TIV and score on the MBI. In this study, we adopted the support vector machine (SVM) as the classifier for building the predictive model, and its kernel function makes use of hyper-plane for separating the cases with or without PSD. In view of the relatively small sample size, *k*-fold cross-validation method (Abdel-Nasser et al., 2015; Uddin et al., 2017; Varoquaux et al., 2017), with *k* = 5, was employed for testing the predictive performance of the SVM model. This method has been adopted in studies which did not involve recruiting an independent sample for model testing. Under this method, all the post-stroke patients were randomly classified into five subgroups. The four subgroups (training set) were used to fit the parameters of each model and the left-out subgroup (test set) was used to estimate the PSD and non-PSD predictive performance. Receiver operating characteristic (ROC) curve using FeAture Explorer (FAE, v0•2•2, https://github.com/salan668/FAE) on Python (3•5•4, https://www.python.org) presented the performances of the SVM model. The accuracy, sensitivity, specificity, positive predictive value (PPV), and negative predictive value (NPV) were computed for the cutoff value that maximizes the Yorden index. All the above processes were implemented

#### 2.6.2 Reproducibility analysis

The leave-one-out method was applied to both the PSD and non-PSD groups (Huang et al., 2016) for testing the reproducibility and robustness of the SVM predictive model. The permutated samples (i.e., 23 PSD vs 33 non-PSD) resulted in a total of 23 two-sample *t*-test images. The voxels which exhibited significant between-group differences across each of the 23 tests were included for computing the reproducibility of the between-group GMV differences.

### 2.7 Data availability

All data needed to evaluate the conclusions are present in the paper and/or the supplementary materials. Additional data related to this paper may be requested from the corresponding author, upon reasonable request.

## 3 Results

### 3.1 Participants' characteristics

Information on the patients’ demographic, medical history, and lesionscan befound in Supplementary Materials. Patients’ brain lesions were overlapped on the right hemisphere. Lesions in the left hemisphere were mirrored across the midsagittal axis and pooled with those in the right hemisphere (Fig. A. 2). The lesions displayed were deemed comparable between the PSD and non-PSD groups as no significant differences were revealed in the distributions o lesions between the left and right hemispheres (PSD: left = 52%, right = 48%; non-PSD: left = 48%, right = 52%) (X^2^ = 3•02, *p* = 0•79) and the different brain regions (PSD: basal ganglia (BG) = 87%, centrum semiovale (CS) = 17%, Insula = 9%; non-PSD: BG = 94%, CS = 6%, Thalamus = 3%) (percentages represent overlap rate of lesion) (X^2^ = 0.08, *p* = 0•40). There were significant between-group differences in the duration of illness and level of education, and scores on the MBI, HAMD, and NIHSS, suggesting that the PSD patients had lower level of activities of daily living performance, higher level of depressive mood symptoms, and higher severity level of stroke than the non-PSD patients (Table 1).

**Table 1:** Comparisons of demographic and medical characteristics, and scores on the clinical assessments between PSD and non-PSD groups.

	PSD patients	Non-PSD patients	*p*-values
Age (years)^a^	59•45 ± 9•74	55•88 ± 11•31	0.22
Sex(male: female)^b^	15:8	25:8	0.77
Duration of illness (months)^a^	12•75 ± 5•32	10•47 ± 3•99	0.07
Level of education (years)^a^	6•09 ± 4•54	8•24 ± 2•53	0.05
Marital status (married: divorced)^c^	22:1	33:0	0.23
TIV^a^	1•42 ± 0•11	1•47 ± 0•12	0.08
BMI^a^	22•48 ± 2•88	23•74 ± 3•48	0.15
MBI^a^	78•00 ± 15•37	91•47 ± 11•18	< 0.001
HAMD^a^	15•00 ± 6•55	4•62 ± 1•79	<0.001
MMSE^a^	25•38 ± 3•90	26•38 ± 2•67	0.25
NIHSS^a^	8•50 ± 2•69	6•21 ± 1•61	< 0.001

Note:.

BMI: body mass index; MBI: Modified Barthel Index; HAMD: Hamilton Depression Scale; MMSE: Mini-Mental State Examination; NIHSS: National Institute of Health stroke scale; TIV: total intracranial volume.

a Mean ± SD, independent *t*-test.

b Fisher exact test.

c Chi-square test.

### 3.2 Between-group difference in GMV

**Fig. 1:**
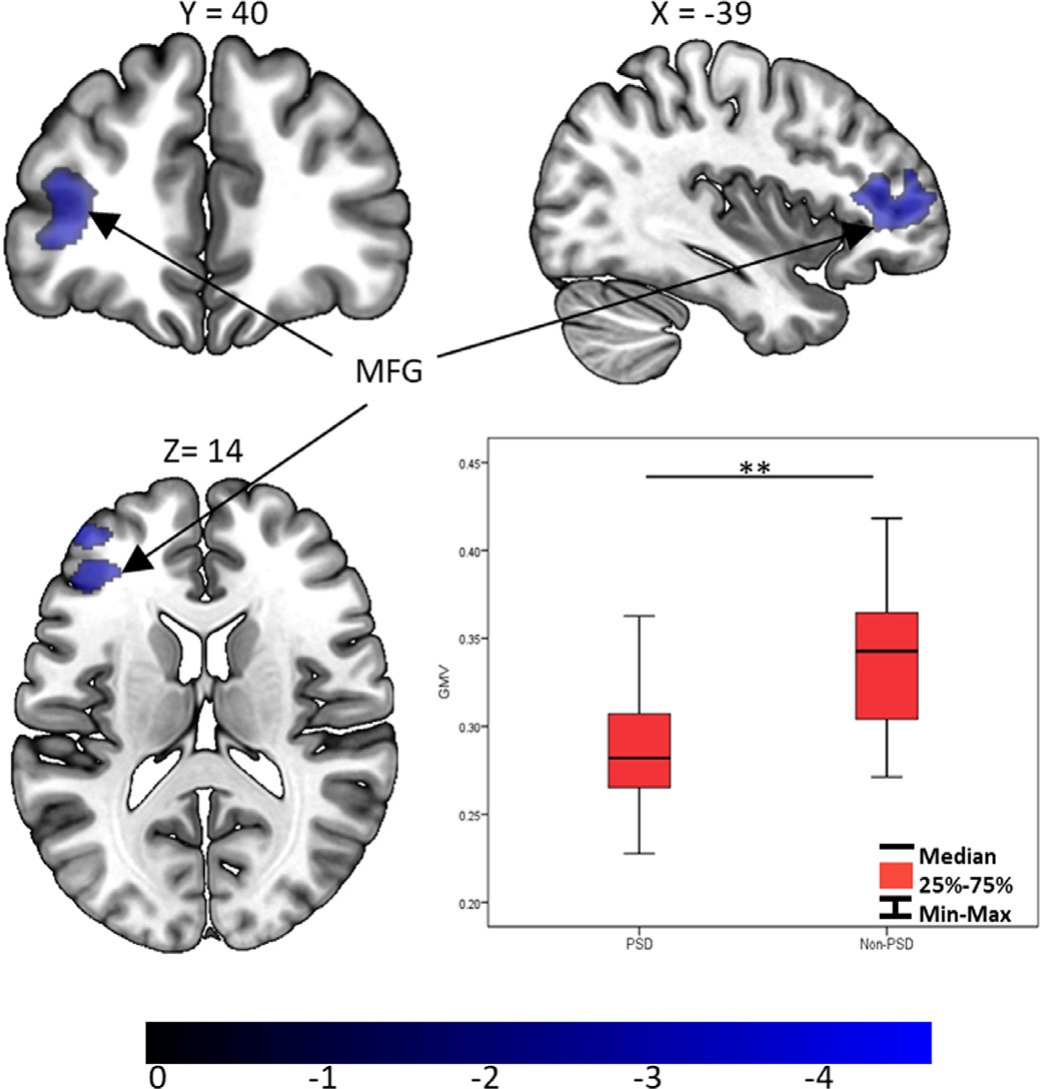
GMV differences between PSD and non-PSD groups. MFG: middle frontal gyrus; GMV: Gray matter volume. Colorbar represents T values. * represents *p* < 0.05; ** represents *p* < 0.01 (two-sample *t*-test).

Compared with the non-PSD group, GMV of the PSD group showed a significant decrease in the left middle frontal gyrus (MFG) (*p* < 0.001, *Cohen's d* = −1•25) (Fig. 1). The large Cohen's d value suggests a large effect size for the MFG result. No other significant results were revealed in other brain structures. In the cluster of left MFG, the inferior frontal gyrus (IFG) accounted for 61% (BA 45 = 41%, BA 47 = 13%, BA 48 = 7%), and the dorsolateral prefrontal cortex (DLPFC) accounted for 39% (BA 46 = 30% and BA 9 = 9%) of the GMV changes.

### 3.3 Correlations between GMV and clinical measures scores

**Fig. 2:**
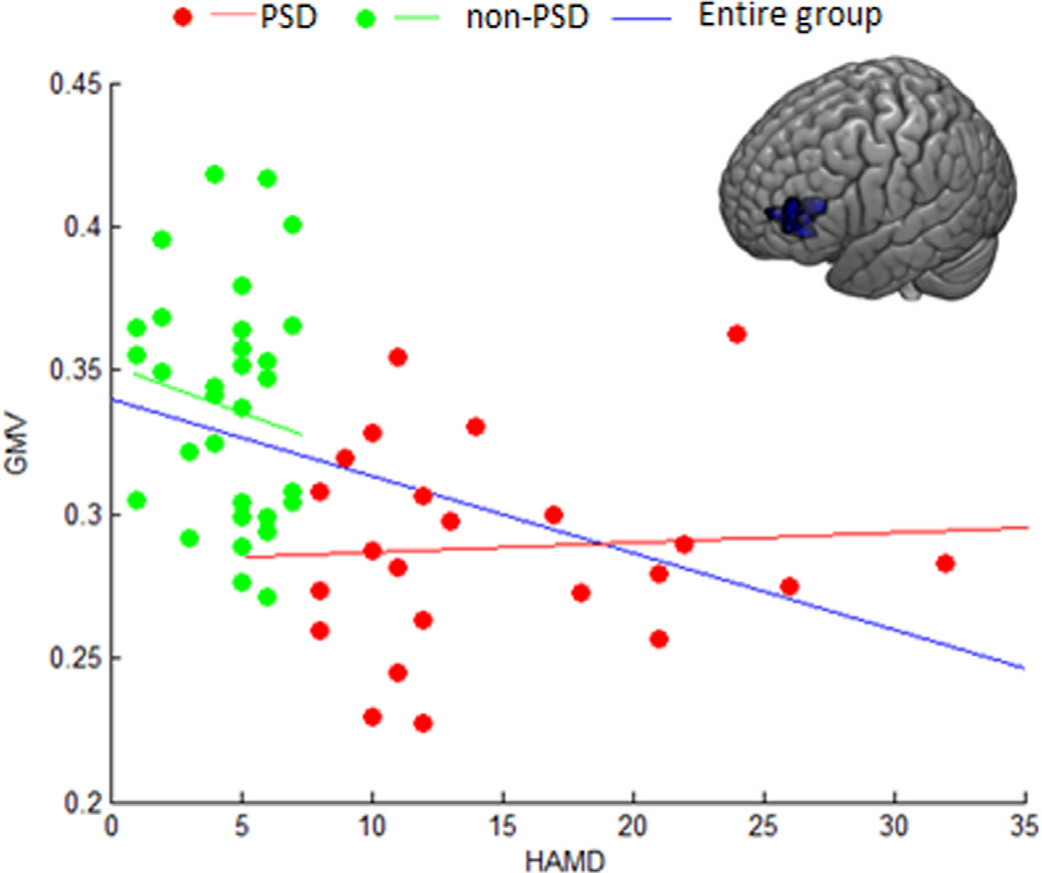
Correlations between GMV of the left MFG and scores of HAMD of patients in the PSD, non-PSD and total groups. MFG: middle frontal gyrus; GMV: gray matter volume; HAMD: Hamilton Depression Scale.

Partial correlation controlling for age, sex, education, time since stroke, and stroke severity as covariates revealed that the mean GMV in the left MFG was significantly correlated with HAMD scores in the total group (*r* = −0•416, *p* = 0•002) (Bonferroni corrected, *p* < 0•05) (Fig. 2). The correlations however were not statistically significant and in fact very low when the total group was divided into the PSD (*r* = −0•089, *p* = 0•73) and the non-PSD groups (*r* = −0•021, *p* = 0•92). The restriction of range analyses were conducted using the method described by Wiberg and Sundström for possible biases in the results due to the potential limited ranges of HAMD scores in each of the PSD and non-PSD groups (Wiberg and Sundström, 2009). The corrected r-values were −0•114 and −0•024 for the PSD and non-PSD groups, respectively. The correlations between the GMVs in the left IFG as well as the left DLPFC and the score of HAMD were not statistically significant. All other correlations between the mean GMV in the MFG and scores of the clinical assessments were non-significant.

### 3.4 SVM and reproducibility

**Fig. 3:**
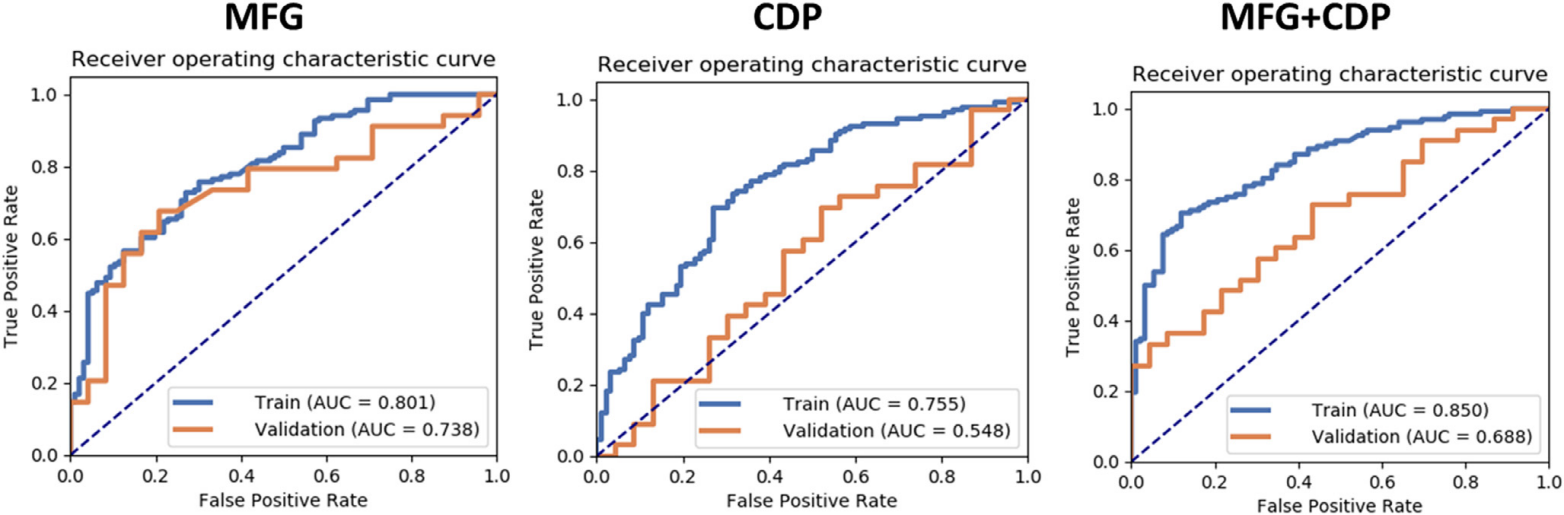
Receiver operating characteristic curves for using MFG, CDP, or MFG+CDP features for classification of PSD versus non-PSD patient memberships. MFG: middle frontal gyrus; CDP: clinical and demographic parameters. Orange curve represents the ROC curve of the validation data set, and blue curve represents the ROC curve of the training data set.

The GMV of the MFG and the significant clinical and demographic parameters (CDP) (age, sex, education level, duration of illness, TIV and MBI) were entered into the model for the SVM analyses (Table 2 and Fig. 3). The MFG model obtained an AUC value of 0•80, with satisfactory sensitivity (0•73) and specificity (0•73). The combined model of MFG+CDP further increased the AUC value to 0•85, which is the highest among all models on the validation data sets. This combined GMV+CDP model yielded a slightly lower sensitivity (0•70) but a higher specificity (0•88) than those of the GMV alone. High level of reproducibility of the MFG was observed in the results of the between-group comparisons (Fig. A. 3).

**Table 2:** Results of SVM analysis for classifying patients into PSD versus non-PSD memberships.

Variables	NPV	PPV	Accuracy	Sensitivity	Specificity	AUC	95% CIs
MFG	0•65	0•79	0•73	0•73	0•73	0•80	0•74–0•86
CDP	0.63	0.79	0.71	0.70	0.73	0.75	0.69–0.82
MFG+ CDP	0•68	0•89	0•78	0•70	0•88	0•85	0•80–0•90

Note: MFG: GMV of middle frontal gyrus; CDP: clinical and demographic parameters (age, sex, education level, duration of illness, TIV and scores of MBI); AUC: area under curve; PPV: positive predictive value; NPV: negative predictive value; CIs: confidence intervals.

## 4 Discussion

To the best of our knowledge, this is the first study exploring the relationship between changes in GMVs and depressive mood symptoms in subcortical post-stroke patients. Our results indicate that compared with non-PSD, PSD group showed a significant decrease in the GMV of the left MFG, particularly in the IFG and DLPFC. Despite the GMVs of the left MFG were found to negatively correlated with the patients’ HAMD score, their relationships are found to be small and non-statistically significant within the PSD group. These findings support the notion that the depressive symptoms and hence the PSD diagnosis are likely to only be related to the abnormal changes in the left IFG and DLPFC. This finding is noteworthy as other common neural substrates in the frontolimbic network such as ACG, hippocampus, and amygdala did not show significant changes in the current predictive model. Together with the rather unique depressive symptoms manifested among the PSD patients, specific intervention regimes may be adopted for treating the depressive symptoms in PSD patients.

The MFG has been reported to play a vital role in mediating emotion (Kret and Ploeger, 2015; Rubin-Falcone et al., 2019), motor (Zhao et al., 2018), and cognitive functions (Sacchet et al., 2017). Volumetric changes of the MFG has been reported to alter emotion control processes (Messina et al., 2013). Previous studies reported that patients with depressive mood have decreased GMV in the MFG when compared to healthy individuals (Droppa et al., 2017; Jiang et al., 2018). Other studies explained that changes in the GMV might have triggered synaptogenesis and dendritic dendriticization (Keifer et al., 2015; Wang et al., 2017). Thus, a decrease in the GMV can lead to synaptogenesis and dendritic dendriticization within the MFG, resulting structural changes and leading to emotion regulation dysfunction, as reflected by the patients’ depressive symptoms. Right hemispheric post-stroke patients were reported to have increased GMV in the right MFG two weeks after onset, and this increase is associated with improvements in motor functions (Wang et al., 2018). Another longitudinal study revealed that, when compared to those in the acute phase, post-stroke patients in the chronic phase showed greater increases in the MFG's GMV (Cai et al., 2016). Findings of these studies indicate that the MFG appears to play a key role in the functional recovery after stroke, and increases in its GMV suggest neural regeneration after the lesions.

In this study, we reported a significant decrease in GMV of the MFG in the PSD patients. The result of the MFG is unique in two ways. First, this finding is rather different from the results revealed by Shi et al. (2017) that the prefrontal involvements were in the orbitofrontal cortex and ACG. Second, the changes in the MFG but not in other frontolimbal neural substrates such as orbitofrontal cortex, ACG, pre-supplementary motor cortex, hippocampus and amygdala (Guo et al., 2015; Monkul et al., 2007) offers plausible explanation to the unique depressive symptoms manifested in PSD patients.

When compared with Shi et al. (2017), all the PSD patients sampled in this study did not have lesions in the frontolimbic network. The changes found in the MFG's GMV resulting in its hypoactivity plausibly suggest that PSD can be associated with a systemic change after the insult to the brain. The consequence is the reduced prefrontal inhibition on the activities of the limbic system resulting in hyperactivity in emotional processing (Mayberg et al., 1999). Our finding in the MFG but not in other neural substrates within the frontal cortex can also explain the observations made by Shi et al. (2017) on the decreased functional connectivity among the prefrontal, cingulate and motor cortices in PSD patients. Future brain imaging and clinical researches are required to investigate these relationships.

The decrease in the MFG's GMV but not in other neural substrates within the frontolimbic network also offer plausible explanations to the unique PSD symptoms revealed by Kim (2016). The common symptoms are depressed mood, anhedonia, loss of energy, decreased concentration, and psychic retardation. Among them, the reduced appetite, psychomotor retardation, and fatigue were reported to be the symptoms best differentiated PSD from non-PSD patients (de Coster et al., 2005). These symptoms are somatic/affective rather than cognitive/affective in nature (Kupper et al., 2012). Abnormalities in the IFG were related to problems with the understanding emotional status and intention (Rota et al., 2009; Tu et al., 2018) and inhibiting appraisal of negative affect (van Tol et al., 2010). Hypoactivity in the left DLPFC was reported to attribute to difficulty in making appropriate emotional judgment (Grimm et al., 2008) and regulate responses to negative emotional stimuli (Fitzgerald et al., 2008). The medial prefrontal cortex-dependent functional coupling was found to be related to the anhedonia symptom (Janes et al., 2018). Besides the unique role of MFG in the prefrontal cluster, the connectivity between the prefrontal and limbic systems in the frontolimbic network may attribute to these symptoms. Connectivity abnormalities between the prefrontal and limbic systems has been shown to account for the psychopathology in MDD (Bennett, 2011; Hultman et al., 2016). In particular, the limbic system forms cortico-dependent circuits by means of monosynaptic connections with the prefrontal cortex (Oh et al., 2014). These circuits are for regulating emotional responses and affective states (Phillips et al., 2003) and psychomotor activity (Kim et al., 2015). Taken together, the involvement of the MFG but not the ACG and orbitofrontal cortex in the frontolimbic network suggests that the PSD patients might not have had problems with processing of emotional-salient information (ACC) (Stuhrmann et al. 2011), and blunting of emotional affect and impaired social functioning (Bremner et al., 2002, Zald and Kim, 2001) and insomnia (Yu et al., 2018) (orbitofrontal cortex).

Antidepressant remains to be a common pharmacological intervention for treating the depressive symptoms among PSD patients. However, reports on its treatment efficacy have not been consistent. An earlier systematic review concluded that antidepressants improved depressive symptoms in PSD patients, but may have resulted in higher remission rates (Hackett et al., 2008). Another review reported that antidepressants were only marginally superior to the placebo (Chen and Guo, 2006), despite a more recent one reported an opposite finding that antidepressants pharmacological therapies reduced the depressive symptoms (Allida et al., 2020). Other studies reported positive effects of the selective serotonin-reuptake inhibitor on alleviating the somatic/affective symptoms such as sleep and anhedonia in depressive patients (Greco et al., 2004; Rolle et al., 2020). Besides, non-pharmacological interventions such as excitatory repetitive transcranial magnetic stimulation (rTMS) over the left DLPFC (Gu and Chang, 2017) or anodal left/cathodal right DLPFC transcranial direct current stimulation (tDCS) (Valiengo et al., 2017) have been shown to reduce depressive symptoms in PSD patients. Future study will need to explore the treatment effects if stimulations are to be applied on the left IFG as well as their specificity to reduce the somatic/affective symptoms commonly manifested among PSD patients.

There are several limitations in this study which limit the power of the analysis as well as generalization of the findings. First, the sample size of post-stroke patients was relatively small. Desipte the k-fold cross-validation method was used to test the performance of the predictive model, future study should use an independent sample for the model training and testing. A larger sample size would improve the power of the analysis, particularly, the correlations between the GMV and the depressive mood symptoms, and the construction of the prediction model for classifying patients into PSD and non-PSD groups. Second, as all the PSD patients had sub-cortical lesions, GMV findings may not necessarily be generalized to those who had other brain lesions. Third, the cross-sectional design in this study did not allow for a conclusion to be drawn on the causes of the structural changes in the frontolimbic circuit. Therefore, how such changes contribute to depressive mood remains speculative. Future study employing a prospective design on a larger sample size and multiple brain lesion groups can facilitate better understanding of the mechanisms underlying depressive mood disorder in post-stroke patients. Last but not least, MMSE was used to measure the cognitive impairments of patients in this study. MMSE, however, was not designed specifically for catering the brain damage characteristics of all post-stroke patients. The results obtained therefore might not have reflected adequately the cognitive problems of the patients. Readers should be cautious when interpreting the results related to this measure. Future study can adopt clinical measures specific to post-stroke patients such as the Chinese (Putonghua) Version of the Oxford Cognitive Screen (Hong et al., 2018).

## 5 Conclusions

This study reports significant changes in the gray matter volume of the left MFG in post-stroke patients diagnosed with depressive symptoms. Our findings on the frontolimbic network change suggest the likelihood that PSD is the result of systemic neural changes independent of the location of the lesions. The hypoactivity in the left IFG and DLPFC as well as the reduced prefrontal inhibition to the limbic system offer plausible explanations on the somatic/affective symptoms manifested in PSD patients. Pharmacological therapies such as selective serotonin-reuptake inhibitors and non-pharmacological interventions such as rTMS and tDCS should be specific to the affected neural substrates and the unique depressive symptoms for improving their effectiveness for patients suffered from PSD.

## Funding

This study was supported by the Department of Rehabilitation Medicine, Nanjing Drum Tower Hospital and the University Research Facility of Behavioral and Systems Neuroscience (UBSN), The Hong Kong Polytechnic University.

## CRediT authorship contribution statement

**Wenjun Hong:** Conceptualization, Writing - original draft, Writing - review & editing. **Zhiyong Zhao:** Methodology, Software, Visualization, Writing - original draft, Writing - review & editing. **Dongmei Wang:** Data curation. **Ming Li:** Data curation. **Chaozheng Tang:** Writing - review & editing. **Zheng Li:** Data curation. **Rong Xu:** Conceptualization. **Chetwyn C.H. Chan:** Conceptualization, Writing - review & editing.

## Declaration of Competing Interest

The authors declare no potential conflicts of interest.

## Appendix Supplementary materials

Image, application 1
