# Supplementary material for: Altered gray matter volumes in post-stroke depressive patients after subcortical stroke
Source: Neuroimage Clin. 2020 Feb 20;26:102224. doi: 10.1016/j.nicl.2020.102224 (PMC7063237; doi:10.1016/j.nicl.2020.102224)
Supplement: Supplementary file 1 [file mmc1.docx]

**Fig. A. 1** Lesion display for patients participating in the study.


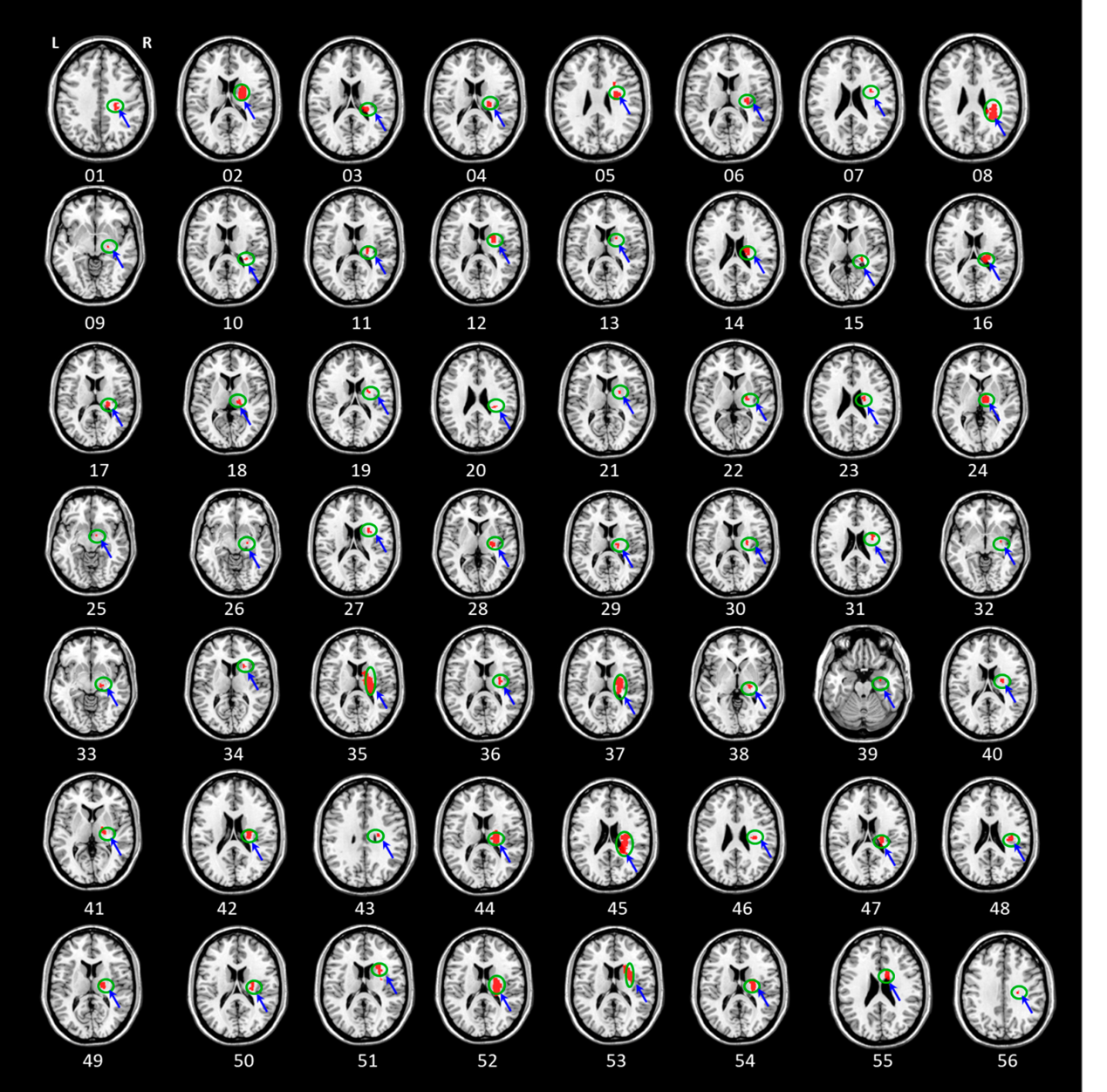


**Note:** Non-PSD group: 01 - 33; PSD group: 34 - 56. The red marker within the green circle pointed by the blue arrowhead shows the lesion location. L: left; R: right.

**Fig. A. 2** Brain lesions of PSD patients (*n* = 23) and non-PSD patients (*n* = 33) are looped on right hemisphere for comparisons.


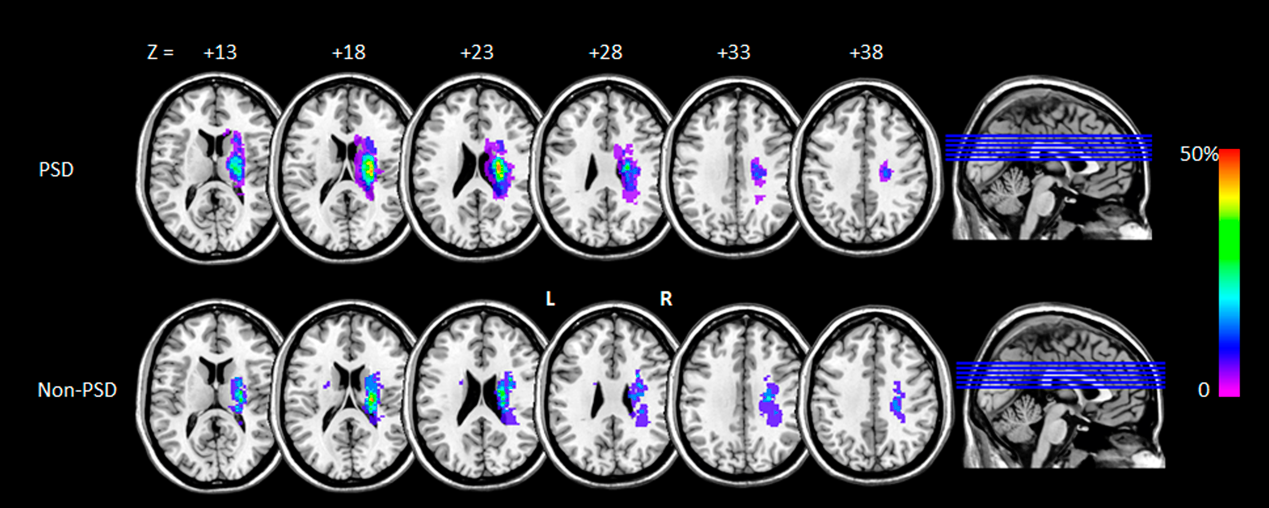


Note: Color displayed in the figure indicates overlap rate of the lesions in each voxel; L: left; R: right.

**Fig. A. 3** Reproducibility analysis of the GMV based on permutated PSD versus non-PSD patient samples for 23 times.

**
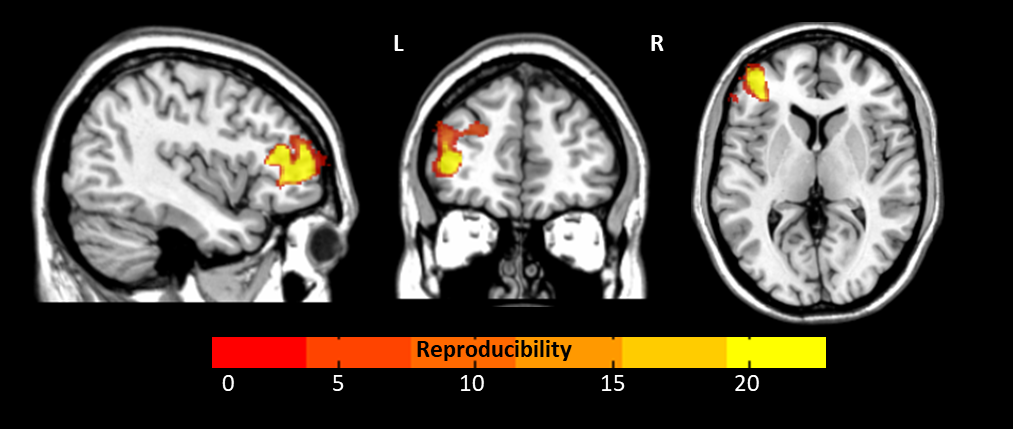
**

**Note:** Color bar indicates the number of the group comparisons showed significant differences in GMV at the voxel level for up to 23 times.

**Table A. 1** Demographic characteristics and clinical assessment results of the PSD patients

| **Case** | **Sex** | **Age**  **(years)** | **Onset duration**  **(months)** | **Lesion location** | **BMI** | **HAMD** | **MMSE** | **NIHSS** | **MBI** |
| --- | --- | --- | --- | --- | --- | --- | --- | --- | --- |
| **PSD group** | | | | | | | | | |
| 01 | F | 48 | 8 | R, BG | 25·00 | 12 | 21 | 6 | 80 |
| 02 | M | 54 | 14 | R, BG | 24·20 | 9 | 26 | 10 | 80 |
| 03 | M | 57 | 8 | L, BG | 18·80 | 13 | 28 | 6 | 90 |
| 04 | F | 55 | 13 | L, BG | 27·30 | 10 | 24 | 4 | 95 |
| 05 | M | 52 | 9 | L, BG | 20·20 | 14 | 24 | 6 | 70 |
| 06 | M | 74 | 14 | R, Insula | 24·20 | 11 | 27 | 7 | 90 |
| 07 | M | 58 | 15 | R, BG | 24·20 | 11 | 29 | 10 | 90 |
| 08 | M | 70 | 11 | L, BG | 22·00 | 17 | 24 | 12 | 60 |
| 09 | M | 53 | 16 | R, BG | 20·20 | 12 | 20 | 8 | 28 |
| 10 | F | 67 | 8 | L, BG | 18·70 | 10 | 24 | 9 | 80 |
| 11 | M | 74 | 33 | R, BG | 21·90 | 8 | 31 | 5 | 54 |
| 12 | F | 60 | 14 | R, CS | 22·10 | 10 | 29 | 6 | 90 |
| 13 | M | 52 | 11 | R, BG | 25·00 | 11 | 28 | 9 | 85 |
| 14 | F | 64 | 10 | L, BG | 22.10 | 18 | 26 | 10 | 80 |
| 15 | M | 72 | 17 | R, BG | 23·90 | 12 | 28 | 6 | 85 |
| 16 | M | 49 | 10 | L, BG | 20·80 | 8 | 33 | 6 | 95 |
| 17 | M | 61 | 9 | L, BG, CS | 24·80 | 8 | 29 | 7 | 85 |
| 18 | F | 73 | 14 | R, BG | 24·70 | 21 | 24 | 11 | 70 |
| 19 | M | 60 | 20 | R, BG | 22·60 | 22 | 19 | 12 | 70 |
| 20 | M | 33 | 11 | L, BG, Insula | 21·50 | 24 | 21 | 10 | 90 |
| 21 | F | 65 | 11 | L, BG | 20·80 | 21 | 18 | 14 | 70 |
| 22 | F | 63 | 10 | L, CS | 17·50 | 26 | 22 | 11 | 65 |
| 23 | M | 59 | 9 | L, BG, CS | 29·00 | 32 | 25 | 12 | 90 |

**Note:** M: male; F: female; L: left; R: right; BG: basal ganglia; CS: centrum semiovale; BMI: Body Mass Index; HAMD: Hamilton Depression Rating Scale; MMSE: Mini-Mental State Examination; NIHSS: National Institute of Health Stroke Scale; MBI: Modified Barthel Index.

**Table A. 2** Demographic characteristics and clinical assessment results of the non-PSD patients

| **Case** | **Sex** | **Age**  **(years)** | **Onset duration**  **(months)** | **Lesion location** | **BMI** | **HAMD** | **MMSE** | **NIHSS** | **MBI** |
| --- | --- | --- | --- | --- | --- | --- | --- | --- | --- |
| **Non-PSD group** | | | | | | | | | |
| 01 | M | 76 | 9 | R, BG | 21·50 | 6 | 28 | 5 | 100 |
| 02 | M | 40 | 12 | R, BG | 23·50 | 5 | 27 | 5 | 100 |
| 03 | M | 58 | 18 | R, BG | 25·30 | 5 | 26 | 5 | 95 |
| 04 | M | 65 | 9 | L, BG | 23·90 | 6 | 25 | 7 | 100 |
| 05 | M | 56 | 8 | R, BG | 19·40 | 6 | 17 | 6 | 100 |
| 06 | F | 66 | 10 | L, BG | 27·80 | 5 | 26 | 6 | 85 |
| 07 | M | 57 | 21 | L, BG | 25·10 | 4 | 27 | 9 | 95 |
| 08 | M | 47 | 18 | R, BG | 28·00 | 4 | 27 | 9 | 60 |
| 09 | M | 66 | 6 | R, BG | 20·20 | 5 | 28 | 6 | 95 |
| 10 | M | 44 | 7 | L, BG | 27·50 | 1 | 26 | 4 | 100 |
| 11 | M | 49 | 15 | L, BG | 20·10 | 5 | 24 | 6 | 65 |
| 12 | M | 55 | 10 | R, BG | 23·90 | 5 | 25 | 5 | 100 |
| 13 | F | 57 | 9 | R, BG | 22·30 | 6 | 30 | 5 | 100 |
| 14 | F | 58 | 17 | L, BG | 18·70 | 2 | 22 | 6 | 80 |
| 15 | M | 56 | 11 | R, BG | 23·90 | 7 | 29 | 7 | 85 |
| 16 | F | 51 | 15 | L, BG | 22·60 | 1 | 26 | 7 | 70 |
| 17 | M | 34 | 13 | L, BG, | 26·40 | 6 | 30 | 5 | 100 |
| 18 | M | 39 | 9 | R, BG | 20·20 | 6 | 26 | 7 | 90 |
| 19 | M | 45 | 5 | R, BG | 24·40 | 4 | 25 | 6 | 95 |
| 20 | M | 50 | 11 | L, BG | 28·30 | 2 | 30 | 7 | 100 |
| 21 | F | 76 | 15 | R, BG | 21·50 | 6 | 26 | 5 | 90 |
| 22 | M | 65 | 8 | L, BG | 24·50 | 7 | 26 | 9 | 80 |
| 23 | F | 86 | 12 | L, BG, CS | 31·20 | 5 | 29 | 6 | 100 |
| 24 | M | 58 | 9 | R, BG | 18·40 | 7 | 25 | 5 | 90 |
| 25 | M | 72 | 8 | R, BG | 32·70 | 3 | 25 | 10 | 95 |
| 26 | F | 60 | 8 | L, BG | 20·80 | 5 | 26 | 4 | 100 |
| 27 | M | 56 | 7 | R, BG | 25·40 | 1 | 30 | 5 | 100 |
| 28 | M | 50 | 8 | L, BG | 19·30 | 2 | 23 | 6 | 100 |
| 29 | M | 42 | 6 | L, BG | 25·70 | 4 | 28 | 4 | 95 |
| 30 | M | 54 | 7 | R, BG | 20·80 | 5 | 26 | 4 | 95 |
| 31 | M | 62 | 10 | L, BG | 21·00 | 3 | 26 | 6 | 100 |
| 32 | F | 50 | 6 | R, CS | 23·70 | 5 | 30 | 7 | 95 |
| 33 | M | 52 | 8 | L, Th | 23·00 | 7 | 29 | 9 | 70 |

**Note:** M: male; F: female; L: left; R: right; BG: basal ganglia; CS: centrum semiovale; Th: thalamus; BMI: Body Mass Index; HAMD: Hamilton Depression Rating Scale; MMSE: Mini-Mental State Examination; NIHSS: National Institute of Health Stroke Scale; MBI: Modified Barthel Index.
